# Substantial Fat Loss in Physique Competitors Is Characterized by Increased Levels of Bile Acids, Very-Long Chain Fatty Acids, and Oxylipins

**DOI:** 10.3390/metabo12100928

**Published:** 2022-09-30

**Authors:** Heikki V. Sarin, Juha J. Hulmi, Youwen Qin, Michael Inouye, Scott C. Ritchie, Susan Cheng, Jeramie D. Watrous, Thien-Tu C. Nguyen, Joseph H. Lee, Zhezhen Jin, Joseph D. Terwilliger, Teemu Niiranen, Aki Havulinna, Veikko Salomaa, Kirsi H. Pietiläinen, Ville Isola, Juha P. Ahtiainen, Keijo Häkkinen, Mohit Jain, Markus Perola

**Affiliations:** 1 THL-Finnish Institute for Health and Welfare, FI-00271 Helsinki, Finland; 2 Research Program for Clinical and Molecular Metabolism, Faculty of Medicine, University of Helsinki, FI-00100 Helsinki, Finland; 3 Neuromuscular Research Center, Faculty of Sport and Health Sciences, University of Jyväskylä, FI-40014 Jyväskylä, Finland; 4 Cambridge Baker Systems Genomics Initiative, Baker Heart and Diabetes Institute, Melbourne, VIC 3004, Australia; 5 School of BioSciences, University of Melbourne, Melbourne, VIC 3010, Australia; 6 Cambridge Baker Systems Genomics Initiative, Department of Public Health and Primary Care, University of Cambridge, Cambridge CB2 0SR, UK; 7 British Heart Foundation Centre of Research Excellence, University of Cambridge, Cambridge CB2 1TN, UK; 8 National Institute for Health Research Cambridge Biomedical Research Centre, University of Cambridge and Cambridge University Hospitals, Cambridge CB2 0QQ, UK; 9 Department of Cardiology, Smidt Heart Institute, Cedars-Sinai Medical Center, Los Angeles, CA 90048, USA; 10 Departments of Medicine and Pharmacology, University of California, San Diego, CA 92093, USA; 11 Sergievsky Center, Taub Institute and Departments of Epidemiology and Neurology, Columbia University, New York, NY 10032, USA; 12 Department of Biostatistics, Mailman School of Public Health Columbia University, New York, NY 10032, USA; 13 Division of Medical Genetics, Departments of Psychiatry, Genetics & Development, Sergievsky Center, New York State Psychiatric Institute, Columbia University, New York, NY 10032, USA; 14 Department of Internal Medicine, University of Turku, FI-20521 Turku, Finland; 15 Division of Medicine, Turku University Hospital, FI-20521 Turku, Finland; 16 Obesity Research Unit, Research Program for Clinical and Molecular Metabolism, Faculty of Medicine, University of Helsinki, FI-00014 Helsinki, Finland; 17 Obesity Center, Abdominal Center, Endocrinology, Helsinki University Hospital and University of Helsinki, FI-00280 Helsinki, Finland

**Keywords:** weight loss, exercise, visceral fat mass, LC-MS metabolome, bioactive metabolites

## Abstract

Weight loss and increased physical activity may promote beneficial modulation of the metabolome, but limited evidence exists about how very low-level weight loss affects the metabolome in previously non-obese active individuals. Following a weight loss period (21.1 ± 3.1 weeks) leading to substantial fat mass loss of 52% (−7.9 ± 1.5 kg) and low body fat (12.7 ± 4.1%), the liquid chromatography-mass spectrometry-based metabolic signature of 24 previously young, healthy, and normal weight female physique athletes was investigated. We observed uniform increases (FDR < 0.05) in bile acids, very-long-chain free fatty acids (FFA), and oxylipins, together with reductions in unsaturated FFAs after weight loss. These widespread changes, especially in the bile acid profile, were most strongly explained (FDR < 0.05) by changes in android (visceral) fat mass. The reported changes did not persist, as all of them were reversed after the subsequent voluntary weight regain period (18.4 ± 2.9 weeks) and were unchanged in non-dieting controls (*n* = 16). Overall, we suggest that the reported changes in FFA, bile acid, and oxylipin profiles reflect metabolic adaptation to very low levels of fat mass after prolonged periods of intense exercise and low-energy availability. However, the effects of the aforementioned metabolome subclass alteration on metabolic homeostasis remain controversial, and more studies are warranted to unravel the complex physiology and potentially associated health implications. In the end, our study reinforced the view that transient weight loss seems to have little to no long-lasting molecular and physiological effects.

## 1. Introduction

Many athletes and an increasing number of other normal-weight individuals engage in vigorous exercise regimens, follow strict dietary plans, and try to rapidly lose fat mass in an attempt to achieve a more aesthetic appearance [1,2] or to improve performance in sports [3]. Prior to competitions, physique athletes engage in very high-volume exercise training and low-energy availability leading to substantial loss of fat mass [4]. In addition to the possible athletic benefits, as outlined above, caloric restriction and fat loss can also improve markers of cardiometabolic health [5]. Paradoxically, at the same time, caloric restriction and low energy availability in athletes can predispose to adverse modulation of physiology (i.e., menstrual dysregulation, hypothyroidism, hypogonadism, and disturbed immune defense and bone metabolism) [4,6,7]. Thus, following a competition diet, voluntary weight regain back to healthy levels has been considered mandatory and beneficial to restore the potentially disrupted metabolic homeostasis caused by prolonged low-energy availability, intense exercise training, and extremely low fat mass. Although obesity and weight gain, in general, have been considered likely to induce detrimental effects on biomarker profiles and overall health, it is yet to be determined whether weight (re)gain from low levels of fat mass within the normal weight range affects human physiology and health in a similar manner.

Advances in molecular profiling technologies, in particular, high-throughput metabolomics, have made it possible to investigate direct signatures of cell biochemical activities in more detail [8] by assessing circulating metabolites in the serum or plasma, thus potentially revealing previously uncharted health effects of weight loss, exercise training, and dietary interventions. To explore metabolome profiles associated with fat loss and regain, we utilized non-targeted liquid chromatography-mass spectrometry (LC-MS) in a sample of competing and non-competing female physique athletes (*n* = 40) who were able to decrease their fat mass to very low levels during diet while observing only very small changes in fat-free mass [4,5,7]. Our earlier study on the same study population with an NMR-metabolomics platform [5] revealed cardiometabolically beneficial changes in lipoprotein metabolism and inflammation markers in physique athletes after competition preparation leading to fat loss. Compared to NMR metabolomics, the LC-MS approach enables an even more thorough characterization of circulating free fatty acids and their derivatives, oxylipins (such as eicosanoids), bile acids, polar molecules, and other biochemically related metabolites [9,10,11,12], that have been implicated in exercise-related physiology, weight loss, lipid metabolism, and cardiometabolic health [13,14,15,16]. Ultimately, this study aims to characterize in a more detailed manner how body fat mass modulation to low levels and fat mass regain affect human metabolome profiles among physically active normal-weight individuals. Considering previous findings on the NMR-metabolome, we hypothesized that a substantial reduction of body fat mass would be associated with widespread changes in the LC-MS metabolome profile, which in young and healthy physically active individuals would be reversible with fat mass regain.

## 2. Materials and Methods

### 2.1. Study Design and Participants: The Physique Study

**Figure 1:**
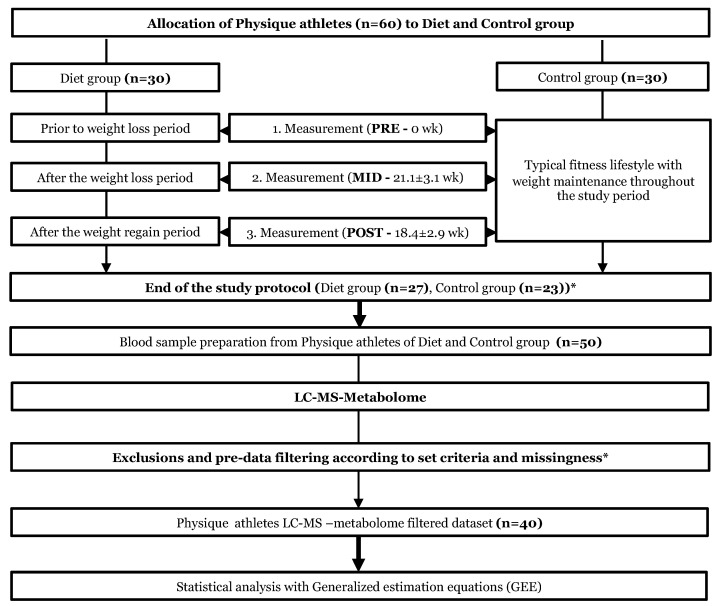
Study design and workflow. Study design and workflow are represented as flowchart to illustrate the whole study protocol. The upper part of the flowchart describes the weight loss and weight regain period of the diet group. In the lower part, we depict the following omics analysis protocol used in the current study. * Of the total 60 participants who started the study, a total of 10 athletes failed to complete the study regimen. In addition, one control did not arrive for baseline testing (PRE), and remaining 9 (*n* = 3 dieters, *n* = 6 controls) participants were excluded due to two reasons (i) short duration of the weight regain period compared with the other participants or (ii) failure to completely follow the study instructions. Additional participants who lacked complete dietary records (*n* = 8) were excluded from the current omics study. In addition, from the *n* = 42, two individuals did not have information on LC-MS metabolomics, resulting in the final sample of *n* = 40 physique athletes that were examined in this study. Furthermore, the sample size varied slightly between different downstream analyses due to incompleteness of omics or phenotype data.

The study cohort consisted of normal weight (BMI: 23.4 ± 1.7 kg/m^2^) female physique athletes (age: 27.5 ± 4.0 years) [4], who aim to achieve a highly-refined aesthetic appearance by reducing body fat levels during a vigorous ~4–5-month progressive competition diet routine, followed by a weight regain period during which energy intake, exercise, and body fat mass levels are restored back to healthy levels (Figure 1). Prior to the study, from a pool of volunteers (*n* = 184), the preliminary requirements were fulfilled by 44 diet and 70 randomly chosen control group candidates, who were sent an online pre-study questionnaire [4]. The diet group size was 30 participants with the inclusion criteria of: (i) age 20–38, (ii) BMI 20–27, (iii) no prevalent diagnosed chronic disease, (iv) no prescribed medication (excluding contraception), and (v) individuals with at least 2 years of resistance training experience. An equal number of participants (*n* = 30) were chosen for the control group by matching those in the diet group based on age, BMI, and a similar level of minimum training background from the pre-study questionnaire. The study subjects were given comprehensive explanations regarding the study design, protocols, and adverse effects of the diets and were monitored throughout the study. Participants were able to withdraw from the study at any time (without providing a reason), and the participants were able to confidentially report to the study MD. An in-depth description of the study design, participants, recruitment, and phenotyping methods was reported previously [4].

As shown in Figure 1, the participating athletes were measured at three time points: (1) baseline measures prior to the weight loss regimen (**PRE**); (2) measures after the diet period, which lasted 21.1 ± 3.1 weeks (**MID**); and (3) measures after the weight regain period, which lasted 18.4 ± 2.9 weeks (**POST**). Participants in the diet group were engaged in rigorous exercise training and lowered energy intake resulting in significant fat mass loss before the competition (**PRE-MID)**, after which they recovered to normal levels of body weight and fat by increasing energy intake and reducing the level of exercise during the subsequent weight regain period (**MID-POST)**. In contrast, participants in the control group were instructed to maintain their typical weight and usual fitness lifestyle, including regular exercise and a healthy diet, and to maintain aesthetic body fat levels while increasing or maintaining muscle mass [4] throughout the study period (**PRE-MID-POST**). At the three time points, participants in both groups went through a series of anthropometric measurements and physical performance tests. The current study on LC-MS metabolomics is a sub-study of a larger Physique Athlete Study conducted by the University of Jyväskylä. A detailed description of the entire study design, participants, and methods has been previously reported [4]. All subjects gave their written informed consent for inclusion before they participated in the study. The study was conducted according to the guidelines of the Declaration of Helsinki, and the protocol was approved by the Ethics Committee of the University of Jyväskylä (approval: 3/2015).

### 2.2. Anthropometric Measurements

In the Physique study, body composition and anthropometrics (including total fat mass, lean mass, and android fat mass) were assessed with several methods, including Dual-energy X-ray absorptiometry (DXA, Lunar Prodigy Advance, GE Medical Systems—Lunar, Madison, WI, USA) and B-mode axial plane ultrasound (model SSD-10, Aloka, Tokyo, Japan) [4]. 

### 2.3. Dietary Information and Physical Activity

All athletes followed their dietary routine during the weight loss and weight regain period, constructed by themselves or their coaches’ instructions. No dietary control or standardizing could be applied due to ethical reasons, but the cohort of physique athletes reported their dietary intakes exceptionally well during the weight loss regimen of the study. The physique athletes reported diet information repeatedly with dietary diary entries on representative days throughout the entire study: at baseline (**PRE**), after the weight loss period (**MID**), and after the weight regain period (**POST**). The food diaries were analyzed by nutrient analysis software (Aivodiet, Flow-team Oy, Oulu, Finland).

For both diet and control groups, the total physical activity level was similarly reported using metabolic equivalent hours per week (MET h/wk). During the study (**PRE**, **MID**, **POST**), the physique athletes reported (i) type, (ii) duration, and (iii) intensity of daily physical activity, from which overall physical activity (MET h/wk) was calculated. The participants followed their own training programs, and they were asked to provide their training diaries throughout the study period (**PRE**, **MID**, **POST**). A more detailed description of dietary intake and physical activity information has been reported previously on the study population [4].

### 2.4. Blood Samples

Fasting plasma samples were collected from the Physique study participants at three time points (**PRE**, **MID**, POST) for omics analyses. Blood was always drawn at the same time of day after at least eight hours of fasting.

### 2.5. Metabolite Extraction and LC-MS METABOLOMICS

Metabolite extraction and measures were performed as previously described [9,10,11,12]. Briefly, plasma metabolites were isolated using protein precipitation with ethanol, followed by isolation using solid phase extraction (SPE) using a Phenomenex Strata-X polymeric 96-well SPE plate. Twenty deuterated internal standards were added for quality analysis. Non-targeted LC-MS analysis was performed using a Phenomenex Kinetex C18 column, coupled to a QExactive orbitrap mass spectrometer equipped with a heated electrospray ionization (HESI) source and collision-induced dissociation (CID) fragmentation. Spectral data were aligned and extracted using in-house custom software, as described [9,10,11,12]. Metabolite identification was confirmed using retention time and MS/MS fragmentation patterns with standards, as described [9,10,11,12]. 

### 2.6. Quality Control and Statistical Analysis of the Metabolome

**Figure 2:**
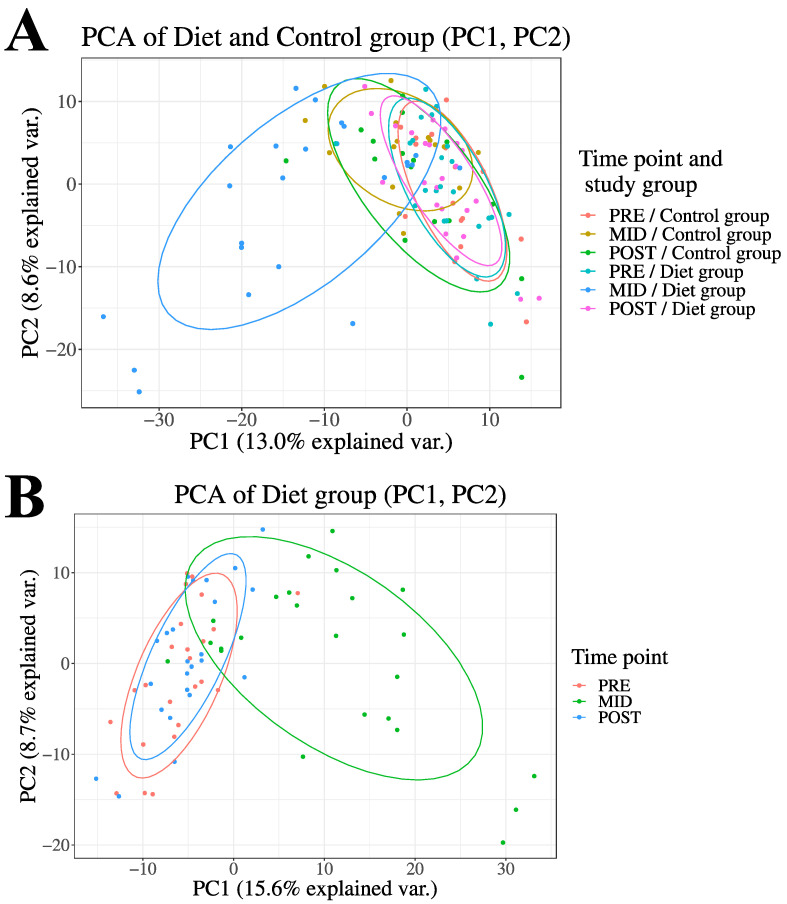
Principal Component Analysis (PCA) of the LC-MS metabolome. Here, we demonstrate overall changes in how the prolonged period of low-energy availability and high volume of exercise training led to substantial fat mass loss (PRE-MID) and subsequent weight regain period (MID-POST) modulated plasma LC-MS metabolome profile (684 metabolite features) in the diet group when compared to controls Panel (**A**). Control group variation during the entire study period (PRE-MID-POST) depicted in two-dimensional PCA plot Panel (**A**). The group and time point annotation is as follows: (1) red = diet group (PRE), (2) green = diet group (MID), (3) blue = diet group (POST), (4) yellow = control group (PRE), (5) turquoise = control group (MID), (6) pink = control group (POST). In panel (**B**), only the diet group-associated changes are demonstrated for clarity of interpretation.

Prior to statistical analysis, the data were preprocessed, normalized, and samples and features with over 20% missing data points were excluded to limit the amount of uncertainty caused by excess missing data. Potential sources of missing data points were as follows (i) the data point not being truly present in the sample, (ii) the data point being below the detection limit of the analysis instrument, (iii) errors made by software in the peak detection, and (iv) alignment phases of the analyses. Second, to dispose of excess variance caused by outliers, feature values were excluded from the analysis if locating more than four standard deviations (SD) from the mean. Third, the remaining missing values (missing and outlier exclusion data points) were imputed using the K-Nearest Neighbour imputation method [17]. Data quality control and filtering were performed separately for both diet and control group time points. Prior to further statistical analyses, principal components analysis (PCA) was performed for the entire LC-MS metabolome dataset (684 metabolite features) to determine the overall structure of the data and changes in the study groups between the time points (Figure 2).

For statistical analysis of the LC-MS metabolome data, we used Generalized Estimating Equations (GEE) [18] with linear links and working independence correlation structures. No transformations were applied to the data, considering the semi-parametric nature of GEE modeling. To investigate whether metabolite feature levels differed across time points, the magnitude of change within diet and control groups was assessed while accounting for between-subject variability and age as possible confounding factors. As a primary analysis, diet and control group were compared across time points (~Time × Group + age) to determine the true effects of the weight loss and regain period. To unravel potential mediators of LC-MS metabolome changes throughout the study (**PRE**, **MID**, **POST**), the primary analysis was further adjusted with android fat mass, total fat mass, energy availability, and physical activity. In the study setting, a randomized controlled trial (RCT) was not feasible, as it is not ethical to conduct an RCT in normal-weight individuals with such a strict diet as these athletes undergo voluntarily. Thus, both the diet and control group were also analyzed separately (i.e., within-group analyses) to (i) confirm findings from primary analyses and ii) evaluate further within-group changes after weight loss and weight regain. *p*-value adjustment for multiple testing was carried out using the Benjamini–Hochberg procedure (FDR) for all analyses conducted on the LC-MS metabolome. R software was used for statistical analyses (version 3.6; https://www.r-project.org, accessed on 1 February 2020). Key packages used for QC and statistical analyses included VIM (version 6.1.0) and geepack (version 1.3-2).

### 2.7. Enrichment Analysis of LC-MS Metabolome

Downstream enrichment analysis of the significant metabolite features was conducted to identify the likely affected biological pathways. For the analyses, the web-based tool, MetaboAnalyst 5.0 (https://www.metaboanalyst.ca, accessed on 18 February 2021) was utilized, as it combines a wide set of integrated databases. Separate lists of significantly increased and decreased metabolites (PRE-MID) were downloaded into the MetaboAnalyst interface to distinguish potential up- and down-regulated biological pathways in the Kyoto Encyclopedia of Genes and Genomes (KEGG) database. It is noteworthy that the ability of MetaboAnalyst to identify LC-MS platform features was limited, as the recognition rate ranged between 45 and 70% of known metabolite features from the LC-MS platform.

## 3. Results

### 3.1. Overview of the Study

As reported previously [4,5,7], the 20-week weight loss period (PRE-MID) consisting of intensive exercise training and a prolonged period of energy deficit yielded significant (*p* < 0.05) changes in the diet group’s body composition: a 12% (−8.1 ± 1.1 kg) reduction in total body weight, a 52% (−7.9 ± 1.5 kg) reduction in total body fat mass, and a 74% (−0.7 ± 1.7 kg) reduction in android fat mass resulting in very low 12.7 ± 4.1 body fat% after the diet (Figure 1; Table S1). These reductions in body weight and fat mass were accomplished by a 33% (−12.3 ± 1.2 kcal/kg/fat-free mass/day) decrease in energy availability and a 17% (9.7 ± 0.5 metabolic equivalent hours per week, METh/wk) increase in the total volume of exercise. No long-term effects on the anthropometric measures were observed (*p* > 0.05) as the subsequent voluntary weight regain period restored body fat mass levels back to baseline levels (MID-POST) (Table S1). In the control group of non-dieting physique athletes, no to minimal changes were observed in the investigated anthropometric measures throughout the study period (PRE, MID, POST) [4,5,7] (Table S1).

### 3.2. Overview of the LC-MS Metabolome Modulation following Substantial Fat Mass Loss and Voluntary Fat Regain

From LC-MS analysis, 684 previously identified metabolite features were used to investigate global metabolic changes in the physique athletes. The principal component analysis (PCA) of the metabolite profiles demonstrated that overall, both of the groups were similar at the baseline (PRE) and after the entire study period (POST), while the weight loss (MID) in the diet group distinctively separated the groups (Figure 2). More specifically, of the investigated features in the LC-MS metabolome profile, a total of 54 significantly altered metabolite features were detected (53 increased, 1 decreased) following the weight loss period (PRE-MID) in the diet group when compared to the controls (Time × Group, FDR < 0.05) (Table 1 and Table S2; Figure 2). After accounting for (i) different isotope adducts associated with the investigated metabolite features and (ii) duplicates—39 unique metabolite features were distinguished (38 increased, 1 decreased) from the overall pool of significantly altered features (Table 1). Among these significantly altered metabolites, most uniform increases (FDR < 0.05) were observed in primary bile acids (BA), very-long-chain fatty acids (VLCFA) and their derivatives, and eicosanoid levels, while a decreased level was only detected in an individual unsaturated FFA, a linoleic acid derivative—γ-linolenate (Table 1). Downstream enrichment analysis of these significant (Time × Group, FDR < 0.05) metabolite features revealed a nominal increase (*p* < 0.05) in *the primary bile acid synthesis pathway* (Figure S1).

**Figure 3:**
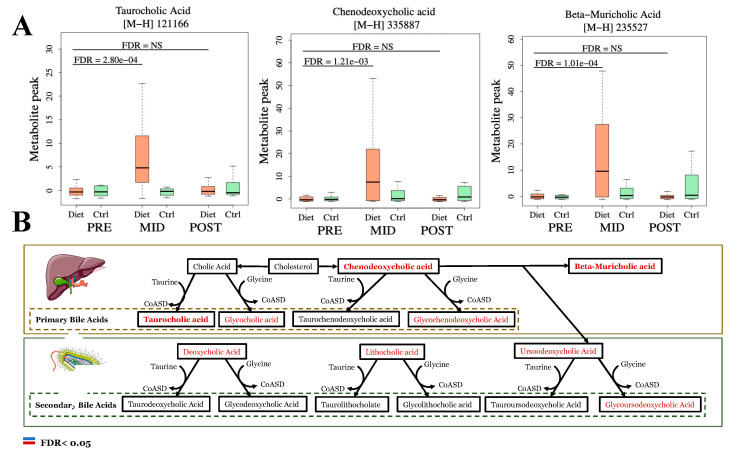
Substantial fat mass loss promotes accumulation of plasma primary bile acid (BA) metabolism intermediates. Bile acid (BA) metabolism pathways related to the generation of primary and secondary BAs are depicted in this figure. In primary and secondary BA metabolism pathways, withing-diet group (~Time) upregulated (FDR < 0.05) metabolites following the weight loss period (PRE-MID) are indicated with red color and bolded where interaction (~Time × Group) was also significant (**Panel B**), and those are also depicted with boxplots (**Panel A**). Metabolites with no clear change in the metabolite level with color black (PRE-MID) (**Panel B**). No decreases were observed in metabolites participating in bile acid metabolism. For boxplot computation, duplicate features of same metabolites were excluded, and features with highest absolute value were selected for plotting.

**Figure 4:**
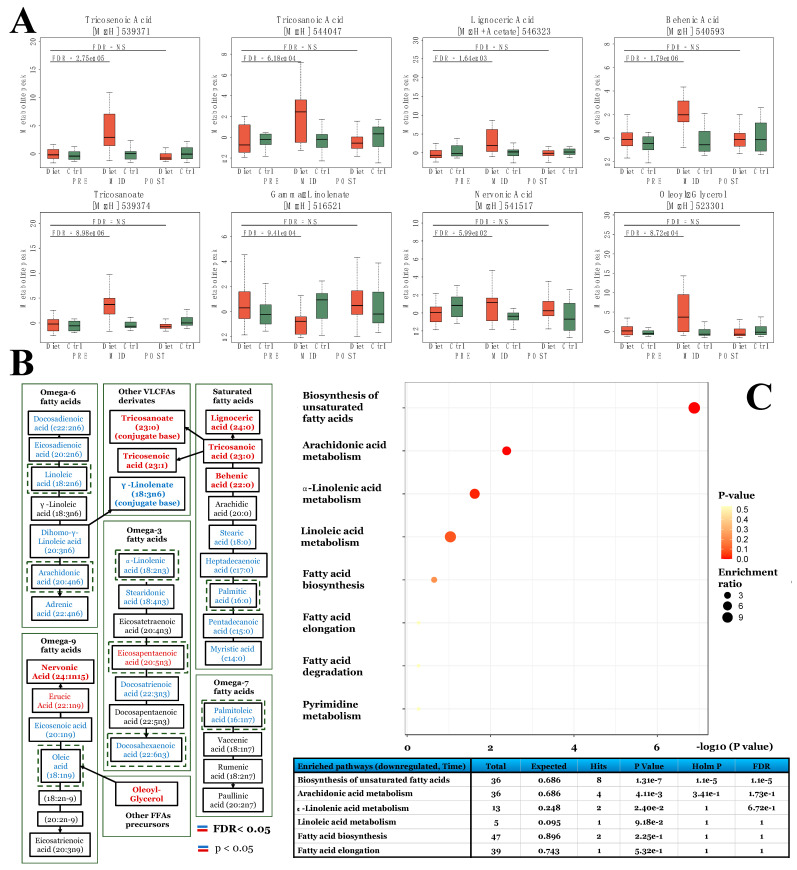
Substantial fat mass loss to low levels promotes accumulation of very-long-chain free fatty acids (VLCFA). Here, we demonstrate how the prolonged period of low-energy availability and high volume of exercise training leading to substantial fat mass loss (PRE-MID in diet group) modulated plasma fatty acid profile. First, FFAs and their metabolites, namely VLCFAs, that were significant in the diet group when compared to controls (Time × Group, FDR < 0.05) are depicted in panel (**A**). Following, fatty acid metabolism pathways related to ω-3, -6, -7, -9, and saturated fatty acids and associated reactions are depicted in panel B. Within unsaturated and saturated fatty acid metabolism pathways panel (**B**), upregulated (FDR < 0.05, red) metabolites are indicated with red color, downregulated (FDR < 0.05, blue) metabolites with blue color, and metabolites with no clear change in metabolite level with black color (PRE-MID). Bold text indicates significance in the interaction (Time × Group) model (Table S2), while normal text significance only in the within-diet group (~Time)-model (Table S3). Lastly, in panel (**C**), results of the enrichment analysis are shown, where, as input, we used all significantly (FDR < 0.05) downregulated metabolite features in the within-diet group analysis after the weight loss period (PRE-MID) (Table S3). Similar to enrichment analysis of the Time × Group findings, only a limited number of these significantly altered unique metabolite features (28 out of 46) were recognized by the MetaboAnalyst platform.

The longitudinal analysis within the diet group only revealed a total of 323 significantly (FDR < 0.05) altered metabolite features following the weight loss period (**PRE-MID**), suggesting a broader perturbation of bioactive lipid molecules in relation to substantial fat mass loss (Table S3). In accordance with the primary results (Time × Group), increased levels of BAs, VLCFAs, and oxylipins (eicosanoid) were observed, which were also accompanied by more uniform decreases in the subclasses consisting of long-chain saturated fatty acids (SFAs) and unsaturated FFAs (Figure 3 and Figure 4; Table 2). Downstream enrichment analysis of these subsets of increased and decreased metabolite features revealed a significant decrease (FDR < 0.05) in *the biosynthesis of the unsaturated FFAs pathway* (Figure 4).

The observed significant changes (**PRE-MID**) did not persist, as all of the reported changes in metabolite features were reversed after the weight regain period (**MID-POST**) in both (i) the comparison analysis between diet and control group and (ii) the within diet group analyses (Figure 2; Table 1 and Table S3). In the within-control group analysis of the LC-MS metabolome, only minor systematic changes were observed throughout the study period, as 42 unique metabolite features were altered significantly (FDR < 0.05) at the halfway point of the study (**PRE-MID**) and only 3 unique metabolite features by the end of the study (**PRE-POST**) (Table S4).

### 3.3. Substantial Fat Mass Loss Promotes Wide Increases in Plasma Bile Acid (BA) Derivatives

Following substantial weight loss (PRE-MID), uniformly increased levels of primary BAs (β-Muricholic-, Chenodeoxycholic-, Taurocholic acid), and a shift towards non-12α-hydroxy dominant BA profile was detected in the diet group when compared to controls (FDR < 0.05) (Table 1; Figure 3). As mentioned above, downstream enrichment analyses supported this observation of upregulated primary bile acid synthesis (Figure S1). These findings were corroborated further by the within-diet group analysis, as increased levels of both primary (taurocholic-, chenodexoycholic-, β-muricholic-, glycocholic acid) and secondary (deoxycholic-, ursodeoxycholic-, glycoursodeoxycholic acid) BAs were suggested following the weight-loss period (Figure 3; Supplementary Table S3). In the within-diet group analysis, in contrast to the elevated levels of BA derivatives, levels of plasma bilirubin were lowered at the end of the weight loss period (FDR < 0.05) (Table S3). No significant changes (FDR > 0.05) among the aforementioned specified BAs or bilirubin were detected in the within-control group analysis (Table S4).

### 3.4. Substantial Fat Mass Loss Achieved through Low-Energy Availability and Physical Activity Promotes Accumulation of Very-Long-Chain Fatty Acids (VLCFAs)

Of the unique 39 significantly altered metabolite features detected by the LC-MS approach in the primary Time × Group analysis, we discovered levels of four unique FFAs to be modulated significantly (FDR < 0.05) following the weight loss period (**PRE-MID**) (Table 1; Figure 4). Specifically, the accumulation of saturated VLCFAs (VLCSFAs) and their derivatives in the plasma was implied through increased levels of (i) lignoceric-, (ii) tricosanoic-, (iii) behenic acid, and (iv) conjugate base of tricosanoic acid (i.e., tricosanoate) (Table 1, Table S2 and Table S3; Figure 4). Contrary to increased levels of VLCSFAs, both the diet and control group FFA profiles were characterized by uniformly diminished levels of medium- to long-chain SFAs (e.g., stearic-, palmitic-, pentadecanoic-, myristic acid) (**MID**) through the within-group analyses (Table 2 and Table S4; Figure 4).

More wide-array accumulation of VLCFA was further supported by increased levels of very-long-chain monounsaturated fatty acids (VLCMUFAs), (i) tricosenoic- and (ii) nervonic fatty acids after the weight loss period in the diet group when compared to controls; although, the latter observation of nervonic fatty acid alteration was mostly driven by attenuation in the levels of the control group (Table 2 and Table S4; Figure 4). No clear evidence of VLCFA accumulation was observed in the within-control group analysis as opposed to the above observations on the diet group (Table S4).

### 3.5. Substantial Weight Loss Achieved by Combined Low-Energy Availability and Physical Activity Is Associated with Diminished Levels of Unsaturated FFAs

Furthermore, from the pool of unsaturated FFA profile, we observed a diminished level (FDR < 0.05) of linoleic acid derivative, γ -linolenate, following the weight loss period in the diet group when compared to controls (Table 1; Figure 4). In addition, reductions in the levels of major ω-3 and ω-6 oxylipin pathway precursors (dihomo-g-linolenic, α-linolenic -, linoleic-, docosahexaenoic-, adrenic-, and arachidonic acid) were observed (FDR < 0.05) (**PRE-MID**), together with wide-array decreases on the levels of unsaturated FFA and their intermediates within the diet group analysis (Table 2; Figure 4). Downstream enrichment analysis of these within-diet group findings revealed a significant decrease (FDR < 0.05) in *the biosynthesis of the unsaturated FFA pathway* (Figure 4). Of the oxylipin pathway precursors, only arachidonic acid demonstrated a similar decrease (FDR < 0.05) in the control group analysis at the halfway point of the study (MID) (Table S4).

### 3.6. Increased Levels of Oxylipins and Eicosanoids Characterize Substantial Weight Loss Achieved by Combined Low-Energy Availability and Physical Activity

The intensive weight loss period (**PRE-MID**) had a recognizable effect on a group of physiologically active lipid compounds called oxylipins (i.e., eicosanoids), which are mainly generated from precursors of ω-3 and ω-6 pathways (Table 1 and Table S3). Uniformly increased levels of 4 characterized (1,12-diHETrE, 13,14-dihydro-15-keto-PGA2, 13S-HpOTrE (γ), 14,15-DiHETE) and 20 uncharacterized unique oxylipin (eicosanoid) features were detected (FDR < 0.05), while none decreased in the diet group when compared to controls after the weight loss period (PRE-MID) (Table 1). The within-diet group analysis further supported the above finding, as the majority (~75%, 84 out of 109) of the significantly (FDR < 0.05) altered unique oxylipin (eicosanoid) features were increased following the weight loss period (PRE-MID) (Table S3). It is noteworthy, however, that despite limited changes observed in the control group metabolome profile, the control group analysis revealed a similar ratio of increases (~74%, 17 out of 23) in the oxylipin profile at the halfway point of the study (MID) (Table S4).

### 3.7. Android Fat Mass Most Strongly Mediates Changes in Free Fatty Acid (FFA), Oxylipin, and Bile Acid Profiles in Female Physique Athletes

Following LC-MS metabolome analysis, we aimed to determine the potential underlying factors mediating the widespread changes in the metabolomic profile of physically active female physique athletes undergoing weight loss and weight regain (Tables S5–S8). Android fat mass most effectively attenuated the observed time-dependent changes in the LC-MS metabolome profiles, as only 16 (15 increased, 1 decreased) of the 54 previously significant metabolite features were observed after model adjustment (Table S5). The inclusion of the android fat mass in the GEE model had the most striking effect on bile acid derivatives, as all of the previously observed changes dissipated. Similar, but not as uniform, effects were observed for oxylipin and FFA profiles, as 75% of the oxylipin and 42% of the FFA changes dissipated. In the end, accounting for total fat mass had similar effects as android fat mass (Table S6), whereas energy availability and physical activity had almost no effect on the LC-MS results (Tables S7 and S8).

## 4. Discussion

This metabolomics study on a unique group of female physique athletes represents an ideal model to explore the effects of fat mass loss to very low levels (i.e., to ~10–15 body fat% by DXA in females) [4] on plasma metabolome profile in normal-weight physically very active individuals. In constructing networks of plasma non-targeted LC-MS metabolomics, our study showed for the first time that intense fat mass loss combined with low energy availability and exercise training results in increased levels of bile acids (BAs), very-long-chain saturated fatty acids (VLCSFAs), and oxylipins (eicosanoids). These widespread changes to the LC-MS metabolome, especially changes in BA profile, were most strongly explained by changes in android and total fat mass, whereas less distinctive effects were observed for physical activity level or energy availability. During the subsequent weight regain period, all of the observed metabolome profile changes were reversed, thereby attenuating doubts about the long-lasting effects of weight cycling in previously physically active normal weight individuals.

Recently, accumulating evidence has characterized BAs as having a central role in improving the regulation of energy metabolism, homeostasis, satiety, body weight regulation, and energy expenditure [19]. Previously, the effects of weight loss on fasting BA levels have been mainly investigated in obese populations following gastric bypass, where after an initial decrease (<1wk post-operation) [20,21], increased levels of BAs have been documented after subsequent weight loss (1–2 years post-operation) [19,20,22,23]. However, other procedures that preserve the integrity of the intestine (i.e., laparoscopic sleeve gastrectomy, gastric banding) have not been associated with elevated BA levels despite similar weight loss and improved insulin sensitivity [20,21,24]. In overweight to obese individuals, contrary to findings from bariatric surgery-induced weight loss, diminished BA pools have been reported after lifestyle-induced weight loss [25,26,27]. Thus, it is plausible that bariatric surgery procedures (especially gastric bypass) lead to altered enterohepatic recirculation of BAs that counteract a weight loss-associated decrease in serum BA levels. Similar to lifestyle-induced weight loss, acute short-term caloric restriction has been shown to halve fasting levels of BAs at first, although follow-up has shown tendencies for BA recovery towards initial baseline levels among obese individuals [28].

Discordant reports of positive [29,30] and negative [31] correlation of BMI with fasting BA levels have also been documented across different BMI categories, together with an absence of reports on clear associations between BA levels and body composition or physical activity [29,32]. Compared to lean, healthy controls, obese insulin-resistant individuals have higher levels of fasting BAs, whereas obese normoglycemic individuals have been characterized with the lowest levels of fasting BAs of these subgroups [20,28,33]. A possible explanation for the above findings and lack of clear association between fasting BAs and BMI or weight loss is that a stronger link seems to exist through glycemic control, as increased levels of fasting BAs have repeatedly been associated with (i) higher levels of visceral fat mass, (ii) insulin resistance, and (iii) diabetes, regardless of overall adiposity [34]. Moreover, phenotypes characterized by insulin resistance have been repeatedly associated with an increased ratio of 12α-hydroxy/non-12α-hydroxy bile acid profile [33]. Although we observed an overall increase in BAs in the present study, a shift towards a non-12α-hydroxy dominant bile acid profile was observed after the weight loss and substantial decrease of fat around the android areas.

Taken together, the existing evidence on how BMI, weight loss, dietary restriction, and exercise training modulate fasting BA levels is somewhat controversial, and there are a number of factors potentially explaining the discordant findings as baseline metabolic status (adiposity, insulin resistance), diet composition, microbiome composition, and fecal excretion rate have all been shown to induce changes in total BA pool making it difficult to distinguish independent effects [28]. To add to the emerging body of evidence, lifestyle-induced weight loss among previously normal-weight physique athletes was shown by us to promote the accumulation of primary BA subsets, a shift towards non-12α-hydroxy dominant bile acid profile, together with a distinct loss of android fat mass, that most strongly explained observed changes in the fasting BA profile. Altogether, we suggest that loss of android fat mass and non-12α-hydroxy dominant bile acid profile may, in part, characterize the putatively advantageous metabolic status of female physique athletes following the weight loss period, although discordant findings from previous [19,20,25,26,27] and our study on the overall level of fasting BA alteration following weight loss need further insight to determine their role among normal-weight individuals. In the past, it has been suggested that moderate levels of BA (e.g., deoxycholic acid) concentrations may be optimal, whereas more extreme levels (low or high) may have deleterious effects. This is why some BAs have been associated with both beneficial and harmful metabolic alterations or health outcomes [35,36,37,38,39,40]. In the end, more studies are warranted to unravel the complex BA-related metabolic homeostasis, networks, their changes after different interventions, and associated health outcomes.

The detailed LC-MS metabolomic platform revealed that fat mass loss to very low levels results in increased levels of saturated VLCFAs, together with diminished levels of unsaturated FFA categories. These findings were, for the most part, in agreement with our earlier study on the same study population with NMR-based analyses, which demonstrated an increase in the amount of overall circulating SFAs together with a reduced degree of unsaturation of the FFA profile following the intense weight loss period [5]. Similar observations have also been documented in dieting obese individuals, where weight loss has resulted in the attenuation of the levels of unsaturated FFAs, which have been shown to correlate with adverse metabolic status contrary to circulating SFAs [41,42]. The aforementioned studies [41,42] have also depicted cardiometabolically positive FFA profiles with lower levels of circulating medium-to-long-chain SFAs, similar to our observations on both within-diet and control group analyses. It has been suggested that discrepancies in FFA profile findings, especially regarding SFAs, are mediated by differences in FFA length and composition [41], as increasing amounts of studies have shown repeatedly that odd chain and VLCSFAs associate with a reduced risk of adverse metabolic outcomes, whereas shorter and even-chain SFAs do not [41,43,44,45,46]. However, some cross-sectional studies have observed the opposite findings between the aforementioned FFA subclasses and health outcomes [47,48], but these discordant findings are likely to be explained by the limitations of cross-sectional studies with small sample sizes and inadequate control for covariates. Taken together, increased levels of circulating VLCFAs, together with attenuated levels of unsaturated FFAs and medium-to-long chain SFAs following lifestyle-induced weight loss, seem to reflect cardiometabolically advantageous FFA profiles even among athletes dieting to very low levels of body fat. 

Despite the association between VLCSFAs and positive health outcomes, studies using cultured myotubes and animal models have demonstrated that exposure to circulating SFAs, especially VLCSFAs, have promoted the formation of ceramides [49,50], while unsaturated FFAs have prevented excess ceramide accumulation stimulated by SFAs. Previously, ceramides have been shown to suppress the electron transport chain and oxidative functions in mitochondria, thus inducing the production of reactive oxygen species—a finding that was subsequently suggested by us through integrative analysis of NMR-metabolomics and leukocyte transcriptomics on the same study population of female physique athletes after weight loss [51]. Interestingly, metabolic states of chronic malnutrition and thus low-energy availability have also been characterized by dysfunction of peroxisomal β-oxidation and the accumulation of VLCFAs [52]. Altogether, we suggest that in addition to reflecting cardiometabolically advantageous FFA profiles [35,37,38,39,40], the increased levels of VLCFAs may also act as a marker for metabolic adaptations, namely attenuated oxidative functions in mitochondria, associated with prolonged periods of low-energy availability and fat mass loss to very low levels. 

In the past, few studies in humans have characterized the effects of weight loss on prostaglandin and oxylipin (eicosanoid) levels focusing on obese individuals [15,53], but none exist in normal-weight individuals. These studies on obese individuals going through weight loss have reported a wide array of effects on oxylipins, mainly towards reduced levels [15]. Promoted proinflammatory cytokine and oxylipin production is considered one key feature of obesity and associated comorbidities [54]; thus, evidence from the above weight loss studies suggests that it is possible to delineate adverse oxylipin profiles in obese individuals through weight reduction [15,53]. Conversely, the metabolome profile of physique athletes by the end of the intense weight loss period was characterized by rather uniform increases in eicosanoid and oxylipin levels, suggesting distinct differences in oxylipin (eicosanoid) metabolism response to weight loss among different categories of baseline weight status. Observed increases in oxylipin (eicosanoid) levels are unlikely to be related to an increased inflammation status, as previously, we showed decreases in the markers of inflammation from these participants [5]. Only the level of arachidonic acid derivative, 11,12-diHETrE, was modulated in a similar, increased manner following weight loss in both dieting physique athletes and the aforementioned study investigating obese individuals by Möller et al. [15]. However, caution should be used when interpreting the effects of oxylipin profile changes between lean and obese individuals since emerging evidence has suggested that physiological roles and health effects of oxylipins may also vary depending on the metabolic context [16].

Similar to the weight status and adiposity level—exercise bouts and exercise training has also been depicted with distinct effects on eicosanoid profiles, where available data indicate that ω-6 and ω-3 oxylipin production is dependent and directly related to the overall volume, intensity, and duration of physical exercise, although the evidence is still limited [16]. The release of oxylipin precursors (PUFAs) from cell membranes can be activated by exercise-induced muscle cell membrane injury, metabolic processes, and signaling pathways promoting increased oxylipin generation. Acute and chronic effects of exercise training have been mainly characterized by increased levels of different oxylipin pathway (COX, LOX, CYP) intermediates [16]; thus, it is plausible that our findings of uniformly increased oxylipin levels following the weight loss period are driven at least partly by the increased level of physical activity observed in female physique athletes. From previously known short- and long-duration exercise training-associated oxylipins [16], the weight loss period among physique athletes resulted in similar modulation of 11,12-diHETrE and PGE2 derivative (13,14-dihydro-15-keto-PGA2), although, the female physique participants were advised to refrain from strenuous exercise training for at least 24 h prior to the measurement [4,16]. Altogether, the current evidence on prostaglandin and oxylipin metabolism modulation following weight loss is limited and equivocal, probably due to differing levels of baseline adiposity and physical activity and whether or not exercise training has been accompanied by weight loss.

Our study has a number of strengths and some limitations. Our study examined the comprehensive system of biological datasets using a longitudinal study design so that it is possible to observe the effects of intensive exercise and diet during the weight loss period followed by the weight regain period. Moreover, our control group was recruited from the same pool of female physique athletes, minimizing potential selection bias. Even though the sample size was modest, repeated measures from a longitudinal design offer sufficient statistical power, as demonstrated by previous omics studies [13]. As a sample of opportunity, given the unsurpassable restrictions that ethics would set on an RCT like this, our study setting is quite unique in reviewing the effects of weight loss for individuals with weight within normal boundaries, thus adding essential information for the ‘normal’ physiology of calorie restriction and exercise in non-obese individuals. However, to confirm our findings, a larger n-size would be warranted in order to validate the adequateness of our sample size and power to capture the biological variance in the measured metabolomic variables. Moreover, although the diet group participants varied systematically and similarly decreased their energy intake, especially from carbohydrates but also from fats, the lack of dietary standardization regarding the type of ingested fats can also be recognized as a limitation of this study, as dietary intake is known to significantly affect the metabolomic profile, especially serum lipid composition. Considering that physique athletes go through repeated cycles of weight loss and weight regain throughout the years of competition preparation and their careers, it is recognized that more longitudinal studies are warranted to examine the effects of long-term intensive exercise with a low-calorie diet and repeated weight loss and weight regain bouts on cardiometabolic markers and health.

In conclusion, a metabolic signature of lean individuals following substantial fat mass loss to very low body fat percentage is characterized by increased levels of plasma primary bile acids, VLCFAs, and oxylipins, together with decreased unsaturated FFAs. Changes in visceral fat associate most strongly with these widespread cardiometabolic changes. This unique group of female physique athletes achieve low levels of fat mass through prolonged periods of high amounts of exercise training and low energy availability, which may explain some of the disparities in responses compared to previous weight loss studies. The physiological significance and health effects of these metabolome profile changes remain to be determined in more detail in the future. In the end, our study reinforced the view that transient weight loss may have little long-lasting molecular and physiological effects as weight regain efficiently ameliorated the detected changes in LC-MS metabolome in previously normal-weight individuals.

## Figures and Tables

**Table 1:** The effect of intensive fat mass loss and fat mass regain on LC-MS metabolome in the diet group compared to control group (Time × Group) of physique study participants.

Annotation and Adduct Information		PRE-MID (Time × Group)			PRE-POST (Time × Group)		
Metabolite Subclass and ID	Matching ID	Estimate	Standard Error	FDR	Estimate	Standard Error	FDR
Bile Acids							
Taurocholic Acid [M-H]	121166	7.80	2.23	**1.74 × 10** ^**−2**^	0.80	1.49	9.51 × 10^−1^
Chenodeoxycholic acid [M-H + Acetate]	335959	10.73	3.70	**4.72 × 10** ^**−2**^	−6.81	3.58	6.77 × 10^−1^
Chenodeoxycholic acid [M-H]	335887	11.36	3.80	**4.05 × 10** ^**−2**^	−7.06	3.71	6.77 × 10^−1^
β-Muricholic Acid [M-H + Acetate]	235588	12.11	3.75	**2.63 × 10** ^**−2**^	−6.42	3.58	6.98 × 10^−1^
β-Muricholic Acid [M-H]	235527	12.34	3.79	**2.63 × 10** ^**−2**^	−6.93	3.63	6.77 × 10^−1^
Free Fatty Acids (FFA)							
Nervonic Acid† [M-H]	541517	2.21	0.61	**1.43 × 10** ^**−2**^	1.54	0.60	6.77 × 10^−1^
Tricosenoic Acid [M-H]	539371	4.15	0.92	**2.53 × 10** ^**−3**^	−0.88	0.53	7.27 × 10^−1^
Tricosenoic Acid [M-H]	542617	3.95	1.05	**1.14 × 10** ^**−2**^	0.35	0.61	9.51 × 10^−1^
Tricosanoic Acid [M-H + Acetate]	542670	1.98	0.61	**2.63 × 10** ^**−2**^	−0.40	0.50	9.26 × 10^−1^
Tricosanoic Acid [M-H]	544047	2.20	0.67	**2.63 × 10** ^**−2**^	−0.37	0.58	9.43 × 10^−1^
Tricosanoic Acid [M-H]	542623	1.93	0.62	**3.22 × 10** ^**−2**^	−0.54	0.52	9.26 × 10^−1^
Lignoceric Acid [M-H + Acetate]	546323	4.19	1.22	**1.79 × 10** ^**−2**^	0.43	0.60	9.26 × 10^−1^
Lignoceric Acid [M-H]	544841	3.19	0.92	**1.79 × 10** ^**−2**^	−0.43	0.72	9.51 × 10^−1^
Lignoceric Acid [M-H]	546284	4.29	1.40	**3.76 × 10** ^**−2**^	0.41	0.66	9.47 × 10^−1^
Behenic Acid [M-H + Acetate]	542079	3.09	1.04	**4.14 × 10** ^**−2**^	−0.33	0.64	9.51 × 10^−1^
Behenic Acid [M-H]	540593	2.17	0.56	**1.01 × 10** ^**−2**^	−0.41	0.49	9.26 × 10^−1^
Behenic Acid [M-H]	542037	3.46	0.91	**1.09 × 10** ^**−2**^	0.08	0.54	9.94 × 10^−1^
Known Eicosanoids							
14,15-DiHETE [M-H + Acetate]	298878	3.56	1.12	**2.77 × 10** ^**−2**^	0.01	0.72	9.98 × 10^−1^
13S-HpOTrE(γ) [M-H]	387101	4.29	1.03	**5.27 × 10** ^**−3**^	−1.57	1.08	7.97 × 10^−1^
13,14-dihydro-15-keto-PGA2 [M-H + Acetate]	253519	5.85	1.94	**3.98 × 10** ^**−2**^	−0.23	0.77	9.78 × 10^−1^
11,12-diHETrE [M-H + Acetate]	350152	5.62	1.60	**1.74 × 10** ^**−2**^	0.78	0.97	9.26 × 10^−1^
11,12-diHETrE [M-H]	355943	2.21	0.73	**3.96 × 10** ^**−2**^	0.63	0.60	9.26 × 10^−1^
Unknown Eicosanoids							
EIC_73	294423	4.10	1.37	**3.98 × 10** ^**−2**^	−0.60	0.80	9.26 × 10^−1^
EIC_71	312209	3.63	1.05	**1.79 × 10** ^**−2**^	0.71	0.68	9.26 × 10^−1^
EIC_69	419015	6.04	1.82	**2.32 × 10** ^**−2**^	1.16	1.35	9.26 × 10^−1^
EIC_69	413523	3.79	1.14	**2.33 × 10** ^**−2**^	0.17	0.73	9.80 × 10^−1^
EIC_69	416464	5.06	1.57	**2.63 × 10** ^**−2**^	0.86	1.02	9.26 × 10^−1^
EIC_64	296371	6.44	1.67	**1.06 × 10** ^**−2**^	0.76	0.70	9.26 × 10^−1^
EIC_62	263579	4.27	1.33	**2.76 × 10** ^**−2**^	−0.10	0.68	9.94 × 10^−1^
EIC_62	253339	7.55	2.49	**3.96 × 10** ^**−2**^	−0.22	0.89	9.80 × 10^−1^
EIC_52	240462	3.17	1.08	**4.32 × 10** ^**−2**^	−0.28	0.54	9.51 × 10^−1^
EIC_51	336280	2.39	0.83	**4.99 × 10** ^**−2**^	−0.42	0.74	9.51 × 10^−1^
EIC_345	397350	3.17	1.02	**3.27 × 10** ^**−2**^	−1.06	0.95	9.26 × 10^−1^
EIC_271	281689	3.98	1.23	**2.63 × 10** ^**−2**^	1.17	0.76	7.78 × 10^−1^
EIC_260	267578	2.39	0.55	**3.23 × 10** ^**−3**^	0.09	0.59	9.94 × 10^−1^
EIC_233	167632	3.58	1.20	**4.06 × 10** ^**−2**^	0.70	0.84	9.26 × 10^−1^
EIC_229	193228	1.68	0.55	**3.76 × 10** ^**−2**^	0.33	0.59	9.51 × 10^−1^
EIC_184	334912	1.95	0.65	**3.98 × 10** ^**−2**^	0.95	0.60	7.42 × 10^−1^
EIC_17	404135	3.22	0.87	**1.14 × 10** ^**−2**^	0.42	0.72	9.51 × 10^−1^
EIC_16	399201	2.25	0.76	**4.16 × 10** ^**−2**^	0.14	0.62	9.80 × 10^−1^
EIC_125	198399	5.09	1.51	**2.11 × 10** ^**−2**^	1.22	1.81	9.43 × 10^−1^
EIC_125	185482	7.97	2.39	**2.31 × 10** ^**−2**^	−0.80	0.79	9.26 × 10^−1^
EIC_121	285160	3.62	1.03	**1.74 × 10** ^**−2**^	0.24	0.63	9.68 × 10^−1^
Novel EIC_9	424299	4.64	1.25	**1.14 × 10** ^**−2**^	−1.43	0.67	6.77 × 10^−1^
Novel EIC_9	422540	3.53	1.19	**4.14 × 10** ^**−2**^	−1.62	0.69	6.77 × 10^−1^
Novel EIC_8	433376	3.72	1.29	**4.78 × 10** ^**−2**^	−0.05	0.69	9.96 × 10^−1^
Novel EIC_5	373373	2.41	0.58	**5.27 × 10** ^**−3**^	0.39	0.58	9.43 × 10^−1^
Novel EIC_28	457606	11.54	3.37	**1.87 × 10** ^**−2**^	−0.43	0.65	9.43 × 10^−1^
Polar Molecules							
Tricosanoate [M-H]	539374	4.66	0.98	**1.29 × 10** ^**−3**^	−0.87	0.62	8.11 × 10^−1^
Oleoyl-Glycerol [M-H]	523301	5.39	1.45	**1.14 × 10** ^**−2**^	−0.98	1.12	9.26 × 10^−1^
Oleoyl-Glycerol [M-H]	522767	4.26	1.36	**3.27 × 10** ^**−2**^	0.45	1.02	9.61 × 10^−1^
γ -Linolenate [M-H]	516521	−2.07	0.56	**1.14 × 10** ^**−2**^	0.08	0.73	9.96 × 10^−1^
Cortisone [M-H + Acetate]	106542	2.42	0.62	**1.01 × 10** ^**−2**^	0.84	0.61	8.11 × 10^−1^
Cortisone [M-H + Acetate]	100714	2.49	0.77	**2.63 × 10** ^**−2**^	1.12	0.61	6.78 × 10^−1^
Putative Molecules							
N-Oleoyl-L-serine	524040	3.22	0.91	**1.74 × 10** ^**−2**^	−0.97	0.56	7.13 × 10^−1^
1-Oleoyl-sn-glycero-3-phosphoethanolamine	463104	3.42	0.98	**1.74 × 10** ^**−2**^	−0.80	0.57	7.97 × 10^−1^

FFA = Free Fatty Acid. EIC = Eicosanoid. FDR = False Discovery Rate. FDR < 0.05 (i.e., **<5 × 10**^**−2**^) are bolded in the table. Results are derived from 4 standard deviation from mean quality-controlled data. Table is ordered based on metabolite feature subclasses. Statistical significance was calculated using Generalized estimation equations (~Time × Group + age). Significance threshold was set to FDR < 0.05, and all significant metabolite features from Time × Group analysis are depicted in Table 1. Analysis was adjusted with age as a covariate. Overall results are depicted in Supplementary Table S2.

**Table 2:** The effect of intensive fat mass loss and fat mass regain on free fatty acid (FFA) and oxylipin precursor profile in the diet group of physique study participants.

Annotation and Adduct Information		PRE-MID (Time)			PRE-POST (Time)		
Metabolite Subclass and ID	Matching ID	Estimate	Standard Error	FDR	Estimate	Standard Error	FDR
Very-long-chain Saturated Fatty acids (VLCSFA)							
Lignoceric Acid [M-H + Acetate]	546323	3.99	1.09	**1.64 × 10** ^**−3**^	0.21	0.39	8.88 × 10^−1^
Lignoceric Acid [M-H]	544841	3.33	0.72	**9.39 × 10** ^**−5**^	−0.1	0.28	9.22 × 10^−1^
Lignoceric Acid [M-H]	546284	4.42	1.30	**3.17 × 10** ^**−3**^	0.21	0.43	9.02 × 10^−1^
Tricosenoic Acid [M-H]	539371	4.14	0.83	**2.75 × 10** ^**−5**^	−0.46	0.26	5.04 × 10^−1^
Tricosenoic Acid [M-H]	542617	2.73	0.96	**1.32 × 10** ^**−2**^	−0.57	0.45	6.34 × 10^−1^
Tricosanoic Acid [M-H + Acetate]	542670	2.19	0.47	**9.23 × 10** ^**−5**^	−0.23	0.31	8.40 × 10^−1^
Tricosanoic Acid [M-H]	542623	2.27	0.48	**6.24 × 10** ^**−5**^	−0.17	0.34	9.00 × 10^−1^
Tricosanoic Acid [M-H]	544047	2.15	0.53	**6.18 × 10** ^**−4**^	−0.10	0.34	9.38 × 10^−1^
Behenic Acid [M-H + Acetate]	542079	3.02	0.88	**2.84 × 10** ^**−3**^	−0.60	0.41	5.74 × 10^−1^
Behenic Acid [M-H]	540593	2.31	0.41	**1.79 × 10** ^**−6**^	0.06	0.27	9.69 × 10^−1^
Behenic Acid [M-H]	542037	3.2	0.79	**6.18 × 10** ^**−4**^	−0.28	0.39	8.40 × 10^−1^
Behenic Acid [M-H]	539090	1.51	0.49	**7.22 × 10** ^**−3**^	0.85	0.80	7.02 × 10^−1^
Long-chain Saturated Fatty acids (LCSFA)							
Stearic Acid [M-H + Acetate]	534421	−1.14	0.35	**4.95 × 10** ^**−3**^	0.46	0.59	8.40 × 10^−1^
Stearic Acid [M-H]	533317	−1.21	0.46	**2.42 × 10** ^**−2**^	−0.91	0.46	4.09 × 10^−1^
Heptadecanoic Acid† [M-H + Acetate] *	534900	−1.98	0.50	**6.67 × 10** ^**−4**^	−1.36	0.63	3.93 × 10^−1^
Heptadecanoic Acid† [M-H + Acetate] *	534128	−1.64	0.42	**8.93 × 10** ^**−4**^	0.11	0.55	9.69 × 10^−1^
Heptadecanoic Acid† [M-H]	532380	−1.29	0.40	**4.51 × 10** ^**−3**^	−0.02	0.42	9.86 × 10^−1^
Heptadecanoic Acid† [M-H] *	534537	−1.44	0.47	**7.30 × 10** ^**−3**^	−0.61	0.49	6.34 × 10^−1^
Heptadecaenoic Acid† [M-H + Acetate] *	529424	−1.86	0.41	**1.20 × 10** ^**−4**^	−0.49	0.57	7.96 × 10^−1^
Heptadecaenoic Acid† [M-H + Acetate] *	532672	−1.54	0.50	**6.57 × 10** ^**−3**^	−0.34	0.66	8.90 × 10^−1^
Heptadecaenoic Acid† [M-H + Acetate]	531646	6.21	2.72	**4.80 × 10** ^**−2**^	0.73	0.59	6.34 × 10^−1^
Heptadecaenoic Acid† [M-H]	529369	−1.83	0.45	**6.39 × 10** ^**−4**^	−0.12	0.55	9.61 × 10^−1^
Heptadecaenoic Acid† [M-H]	531845	−1.16	0.37	**6.36 × 10** ^**−3**^	−0.10	0.45	9.61 × 10^−1^
Palmitic Acid [M-H + Acetate]	531306	−1.63	0.41	**8.72 × 10** ^**−4**^	−0.36	0.48	8.40 × 10^−1^
Palmitic Acid [M-H + Acetate]	534889	−0.72	0.30	**3.87 × 10** ^**−2**^	0.09	0.34	9.46 × 10^−1^
Palmitic Acid† [M-H + Acetate] *	531996	−1.38	0.42	**4.37 × 10** ^**−3**^	−0.04	0.53	9.86 × 10^−1^
Palmitic Acid [M-H]	533569	−0.76	0.32	**3.81 × 10** ^**−2**^	0.46	0.42	6.89 × 10^−1^
Pentadecanoic Acid [M-H]	530326	−1.45	0.45	**5.19 × 10** ^**−3**^	−0.33	0.47	8.48 × 10^−1^
Pentadecanoic Acid [M-H]	528566	−1.15	0.46	**2.95 × 10** ^**−2**^	−0.22	0.51	9.03 × 10^−1^
Pentadecanoic Acid† [M-H + Acetate] *	531478	−2.07	0.45	**9.24 × 10** ^**−5**^	−0.27	0.72	9.22 × 10^−1^
Myristic Acid [M-H + Acetate]	524395	−1.63	0.53	**6.50 × 10** ^**−3**^	−0.34	0.75	9.03 × 10^−1^
Myristic Acid [M-H + Acetate]	522387	−1.74	0.50	**2.45 × 10** ^**−3**^	−0.05	0.58	9.86 × 10^−1^
Myristic Acid [M-H] *	524668	−1.55	0.48	**4.97 × 10** ^**−3**^	−0.33	0.63	8.90 × 10^−1^
Myristic Acid [M-H]	522346	−1.64	0.50	**4.48 × 10** ^**−3**^	−0.2	0.56	9.22 × 10^−1^
Very-long-chain Monounsaturated Fatty acids							
Tricosenoic Acid [M-H]	539371	4.14	0.83	**2.75 × 10** ^**−5**^	−0.46	0.26	5.04 × 10^−1^
Tricosenoic Acid [M-H]	542617	2.73	0.96	**1.32 × 10** ^**−2**^	−0.57	0.45	6.34 × 10^−1^
Tricosanoic Acid [M-H + Acetate]	542670	2.19	0.47	**9.23 × 10** ^**−5**^	−0.23	0.31	8.40 × 10^−1^
Omega-3 Fatty Acids							
Docosahexaenoic Acid (DHA) [M-H + Acetate]	529467	−1.01	0.40	**2.71 × 10** ^**−2**^	0.02	0.38	9.86 × 10^−1^
Docosahexaenoic Acid (DHA) [M-H]	525743	−1.03	0.32	**5.40 × 10** ^**−3**^	0.32	0.37	7.93 × 10^−1^
\ Docosatrienoic Acid [M-H]	532415	−1.5	0.37	**5.70 × 10** ^**−4**^	0.35	0.46	8.40 × 10^−1^
Eicosapentaenoic Acid (EPA) [M-H + Acetate]	523035	1.34	0.35	**1.08 × 10** ^**−3**^	0.13	0.38	9.24 × 10^−1^
Eicosapentaenoic Acid (EPA) [M-H]	523009	1.26	0.36	**2.28 × 10** ^**−3**^	0.02	0.38	9.86 × 10^−1^
Stearidonic Acid [M-H]	488156	−3.39	1.35	**2.84 × 10** ^**−2**^	−2.48	1.28	4.09 × 10^−1^
α-Linolenic Acid [M-H]	523928	−1.15	0.33	**2.50 × 10** ^**−3**^	0.49	0.53	7.76 × 10^−1^
α-Linolenic Acid [M-H]	521670	−1.71	0.52	**4.51 × 10** ^**−3**^	−0.42	0.59	8.48 × 10^−1^
Omega-6 Fatty Acids							
Docosadienoic Acid [M-H]	534554	−1.29	0.34	**1.18 × 10** ^**−3**^	0.32	0.39	8.06 × 10^−1^
Docosadienoic Acid [M-H]	536158	−1.52	0.47	**5.13 × 10** ^**−3**^	−0.95	0.51	4.53 × 10^−1^
Eicosadienoic Acid [M-H]	531992	−1,50	0.32	**9.39 × 10** ^**−5**^	0.23	0.37	8.69 × 10^−1^
Linoleic Acid [M-H]	526174	−1.39	0.38	**1.64 × 10** ^**−3**^	0.04	0.45	9.86 × 10^−1^
Dihomo- γ -linolenic [M-H]	530664	−1.71	0.28	**8.71× 10** ^**−8**^	0.43	0.38	6.79 × 10^−1^
Dihomo- γ -linolenic [M-H]	529407	−1.61	0.35	**1.02 × 10** ^**−4**^	0.20	0.40	8.95 × 10^−1^
Arachidonic Acid [M-H]	529143	−1.42	0.29	**4.21 × 10** ^**−5**^	0.25	0.49	9.00 × 10^−1^
Arachidonic Acid [M-H + Acetate] *	531873	−1.30	0.36	**1.64 × 10** ^**−3**^	0.20	0.38	8.88 × 10^−1^
Adrenic Acid [M-H]	528116	−1.45	0.33	**1.82 × 10** ^**−4**^	1.10	0.51	3.93 × 10^−1^
Adrenic Acid [M-H]	532126	−1.83	0.43	**2.69 × 10** ^**−4**^	−0.26	0.50	8.89 × 10^−1^
Adrenic Acid [M-H]	530967	−1.58	0.39	**5.70 × 10** ^**−4**^	0.40	0.46	7.93 × 10^−1^
Omega-7 Fatty Acids							
Palmitoleic Acid [M-H + Acetate]	526018	−1.35	0.36	**1.27 × 10** ^**−3**^	0.32	0.36	7.93 × 10^−1^
Palmitoleic Acid [M-H + Acetate]	529152	−1.64	0.62	**2.09 × 10** ^**−2**^	−1.13	0.63	4.96 × 10^−1^
Palmitoleic Acid [M-H]	531843	−2.12	0.39	**3.23 × 10** ^**−6**^	−0.18	0.43	9.10 × 10^−1^
Palmitoleic Acid [M-H]	529701	−2.21	0.41	**5.66 × 10** ^**−6**^	−0.31	0.36	7.96 × 10^−1^
Palmitoleic Acid [M-H]	526135	−1.37	0.34	**6.18 × 10** ^**−4**^	0.25	0.32	8.40 × 10^−1^
Omega-9 Fatty Acids							
Nervonic Acid [M-H]	544451	2.01	0.64	**5.89 × 10** ^**−3**^	−0.71	0.43	5.50 × 10^−1^
Nervonic Acid [M-H]	539105	3.15	0.85	**1.43 × 10** ^**−3**^	−0.02	0.38	9.86 × 10^−1^
Docosaenoic Acid† (Erucic Acid) [M-H] *	535834	3.17	0.76	**3.80 × 10** ^**−4**^	0.21	0.50	9.10 × 10^−1^
Eicosatrienoic Acid† [M-H + Acetate] *	534431	−1.54	0.44	**2.29 × 10** ^**−3**^	0.30	0.56	8.88 × 10^−1^
Eicosenoic Acid [M-H]	534395	−1.36	0.38	**1.87 × 10** ^**−3**^	−0.16	0.38	9.03 × 10^−1^
Eicosenoic Acid [M-H]	531994	−1.35	0.38	**2.30 × 10** ^**−3**^	0.35	0.55	8.69 × 10^−1^
Eicosenoic Acid [M-H]	539066	−1.46	0.45	**4.51 × 10** ^**−3**^	−0.12	0.44	9.46 × 10^−1^
Oleic Acid [M-H + Acetate]	531868	−0.94	0.36	**2.42 × 10** ^**−2**^	0.57	0.36	5.50 × 10^−1^
Oleic Acid [M-H]	535786	−1.37	0.39	**2.44 × 10** ^**−3**^	0.09	0.47	9.69 × 10^−1^
Oleic Acid [M-H]	534665	−1.4	0.44	**5.48 × 10** ^**−3**^	0.4	0.59	8.54 × 10^−1^
Oleic Acid [M-H]	531847	−0.96	0.39	**3.00 × 10** ^**−2**^	0.19	0.34	8.88 × 10^−1^

FFA = Free Fatty Acid. EIC = Eicosanoid. FDR = False Discovery Rate. FDR < 0.05 (i.e., **<5 × 10**^**−2**^) are bolded in the table. Results are derived from 4 standard deviation from mean quality-controlled data. Table is ordered based on metabolite feature subclasses. Statistical significance was calculated using Generalized estimation equations (~Time + age). Significance threshold was set to FDR < 0.05, and all significant metabolite features from within diet group analysis and among specified subclasses are depicted in Table 2. Analysis was adjusted with age as covariate. * Denotes metabolite features that were also significant (FDR < 0.05) and modulated in the same direction in the Control group analysis.

## Data Availability

The data presented in this study are available on reasonable request from the corresponding author. The data are not publicly available due to institutional regulations. In order to gain access to these datasets, applications must be submitted to the Finnish Institute for Health Welfare, Helsinki, Finland, according to the terms of data distribution protocols set by the Finnish Institute for Health Welfare, Helsinki, Finland.

## Supplementary Materials

The following supporting information can be downloaded at: https://www.mdpi.com/article/10.3390/metabo12100928/s1, Supplementary material 1 (Tables S1–S8).xlsx (Table S1. Phenotype charasteristics and their changes in the diet and control group participants throughout the study, Table S2. The effect of intensive fat mass loss and fat mass regain on LC-MS-Metabolome in the diet group compared to control group of physique study participants, Table S3. The effect of intensive fat mass loss and fat mass regain on LC-MS-Metabolome in the diet group of physique study participants, Table S4. The effect of eukaloric diet and fitness lifestyle on LC-MS-Metabolome in the control group of physique study participants, Table S5. The effect of intensive fat mass loss and fat mass regain on LC-MS-Metabolome in the diet group compared to control group of physique study participants, when adjusted with android fat mass (DEXA), Table S6. The effect of intensive fat mass loss and fat mass regain on LC-MS-Metabolome in the diet group compared to control group of physique study participants, when adjusted with total fat mass (DEXA), Table S7. The effect of intensive fat mass loss and fat mass regain on LC-MS-Metabolome in the diet group compared to control group of physique study participants, when adjusted with physical activity, Table S8. The effect of intensive fat mass loss and fat mass regain on LC-MS-Metabolome in the diet group compared to control group of physique study participants, when adjusted with energy availability) and Supplementary material 2 (Figure S1).docx (Figure S1. Enrichment analysis indicates altered biological pathways and entities following intensive fat mass loss. In Panel A, we show top pathways enriched with significantly altered (53 upregulated, 1 downregulated, FDR < 0.05) LC-MS metabolite features (PRE-MID in diet group when compared to controls), where color red indicates enrichment of upregulated pathway, whereas blue enrichment of downregulated pathway. From the input of 18 significantly altered known unique metabolite features, MetaboAnalyst recognized only 12 known unique metabolite features that were used to calculate the enrichment analyses results. Subsequently, in Panel B, we show top pathways enriched with significantly increased (FDR < 0.05) metabolite features (PRE-MID in diet group). From the input of 62 significantly increased unique metabolite features, MetaboAnalyst recognized 28 known unique metabolite features that were used to calculate the enrichment analyses results. Moreover, see Figure 4, for the enrichment results of downregulated metabolite features within diet group after the weight loss period. No enrichment analyses were conducted for the weight regain period (MID-POST), or in control group at any timepoint due to limited number of significant metabolites (Table S3,S4).)
