# Supplementary material for: Substantial Fat Loss in Physique Competitors Is Characterized by Increased Levels of Bile Acids, Very-Long Chain Fatty Acids, and Oxylipins
Source: Metabolites. 2022 Sep 30;12(10):928. doi: 10.3390/metabo12100928 (PMC9609491; doi:10.3390/metabo12100928)
Supplement: Supplementary file 1 [file metabolites-12-00928-s001.zip › metabolites-1917399-supplementary.pdf]

A

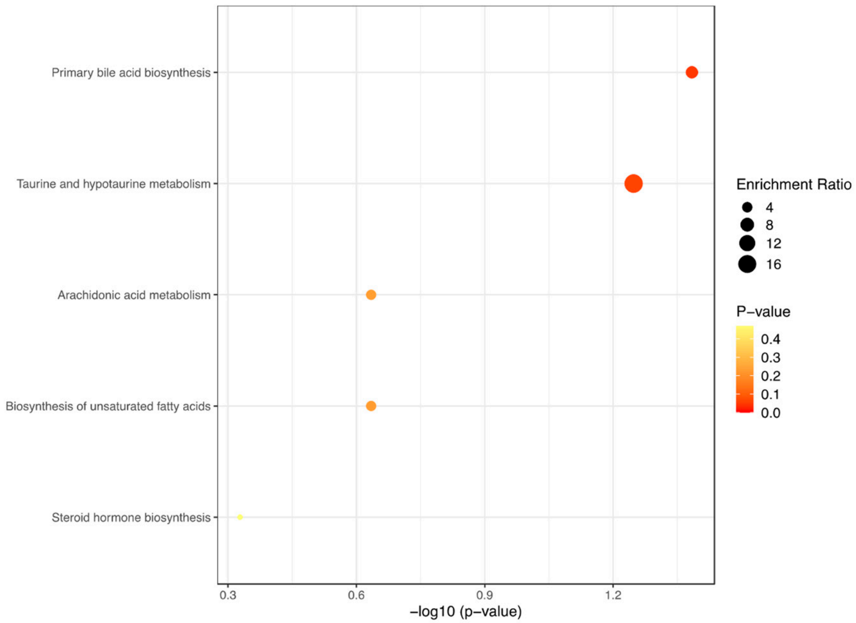

Enriched pathways (Time*Group)	Total	Expected	Hits	P value	Holm P	FDR
Primary bile acid biosynthesis	46	0.332	2	0.0413	1	1
Taurine and hypotaurine metabolism	8	0.0578	1	0.0565	1	1
Arachidonic acid metabolism	36	0.26	1	0.232	1	1
Biosynthesis of unsaturated fatty acids	36	0.26	1	0.232	1	1
Steroid hormone biosynthesis	85	0.614	1	0.47	1	1

B

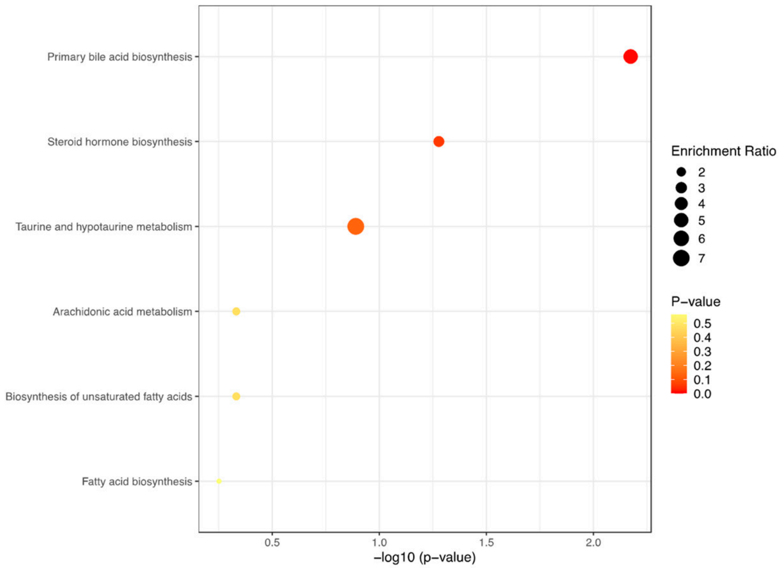

Enriched pathways (upregulated, Time)	Total	Expected	Hits	P value	Holm P	FDR
Primary bile acid biosynthesis	46	0.786	4	0.00671	0.564	0.564
Steroid hormone biosynthesis	85	1.45	4	0.0528	1	1
Arachidonic acid metabolism	36	0.615	2	0.124	1	1
Taurine and hypotaurine metabolism	8	0.137	1	0.129	1	1
Biosynthesis of unsaturated fatty acids	36	0.615	1	0.466	1	1
Fatty acid biosynthesis	47	0.803	1	0.561	1	1

Supplementary Figure S1. Enrichment analysis indicates altered biological pathways and entities following intensive fat mass loss. In Panel A, we show top pathways enriched with significantly *altered* (53 upregulated, 1 downregulated, FDR < 0.05) LC-MS metabolite features (PRE-MID in diet group when compared to controls), where color red indicates enrichment of upregulated pathway, whereas blue enrichment of downregulated pathway. From the input of 18 significantly altered known unique metabolite features, MetaboAnalyst recognized only 12 known unique metabolite features that were used to calculate the enrichment

analyses results. Subsequently, in Panel B, we show top pathways enriched with significantly *increased* (FDR < 0.05) metabolite features (PRE-MID in diet group). From the input of 62 significantly increased unique metabolite features, MetaboAnalyst recognized 28 known unique metabolite features that were used to calculate the enrichment analyses results. Moreover, see Figure 4, for the enrichment results of downregulated metabolite features within diet group after the weight loss period. No enrichment analyses were conducted for the weight regain period (MID-POST), or in control group at any timepoint due to limited number of significant metabolites (Supplementary Table 3-4).
